# Revisiting the Characterization of the Losses in Piezoelectric Materials from Impedance Spectroscopy at Resonance

**DOI:** 10.3390/ma9020072

**Published:** 2016-01-26

**Authors:** Amador M. González, Álvaro García, César Benavente-Peces, Lorena Pardo

**Affiliations:** 1 ETS Ingeniería y Sistemas de Telecomunicación, Campus Sur UPM, Madrid 28031, Spain; cbenavente@ics.upm.es; 2 Instituto de Ciencia de Materiales de Madrid-CSIC, Madrid 28049, Spain; alvarog@icmm.csic.es (Á.G.); lpardo@icmm.csic.es (L.P.)

**Keywords:** piezoelectric materials, ceramics, mechanical losses, dielectric losses, piezoelectric losses, electromechanical resonances, material characterization, impedance spectroscopy

## Abstract

Electronic devices using the piezoelectric effect contain piezoelectric materials: often crystals, but in many cases poled ferroelectric ceramics (piezoceramics), polymers or composites. On the one hand, these materials exhibit non-negligible losses, not only dielectric, but also mechanical and piezoelectric. In this work, we made simulations of the effect of the three types of losses in piezoelectric materials on the impedance spectrum at the resonance. We analyze independently each type of loss and show the differences among them. On the other hand, electrical and electronic engineers include piezoelectric sensors in electrical circuits to build devices and need electrical models of the sensor element. Frequently, material scientists and engineers use different languages, and the characteristic material coefficients do not have a straightforward translation to those specific electrical circuit components. To connect both fields of study, we propose the use of accurate methods of characterization from impedance measurements at electromechanical resonance that lead to determination of all types of losses, as an alternative to current standards. We introduce a simplified equivalent circuit model with electrical parameters that account for piezoceramic losses needed for the modeling and design of industrial applications.

## 1. Introduction

### 1.1. Losses in Piezoelectric Materials

New electronic devices need better components to get better performance and improved functionalities. In the case of devices using the piezoelectric effect, the heart of the device consists of a piezoelectric element: a crystal [1]; but in most cases, poled ferroelectric ceramics (piezoceramics) [2], polymers [3] or composite materials [4]. The crystal structure of piezoelectrics is non-centrosymmetric, but randomly-oriented polycrystals are centrosymmetric. An induced macroscopic non-centrosymmetry is needed for a polycrystal to be piezoelectric. This induced symmetry can be provided by an external electric field (poling) or mechanical action (stretching).

Among the devices commonly using piezoelectric materials, we can find numerous sensors and actuators, which are classically used in telecommunications, medicine or industrial quality control, but new applications in energy storage or energy harvesting are also being developed nowadays.

A piezoelectric material or device can be driven by an electric or mechanical stimulus and responds with both electrical and mechanical reactions.

System losses are defined as the rate of energy provided to the system that cannot be transformed into work. Usually, we call this loss of energy dissipation. This definition needs to be applied, and frequently rewritten, for every process involving energy conversion. Friction, dielectric dissipation, damping, *etc.*, are related to losses in piezoelectric materials.

A piezoelectric material has losses originating from the dielectric response to an electrical field, the mechanical response to applied stress, or its piezoelectric motion, or its strain response to the electric field, or conversely, the charge or voltage generation as a response to the applied stress. Losses in piezoelectric materials result in sample heating or noise production. These effects are detrimental in many applications, and this is why the understanding of loss mechanisms and knowledge of the actual value of the loss in the material becomes a key issue for the design of devices. Furthermore, the control of the loss mechanism is needed to optimize the efficiency of the electromechanical transduction and, consequently, the device performance. For this reason, many authors have treated this topic in ferro-piezoelectric ceramics and related materials [5,6,7,8,9,10,11,12,13,14,15]. To date, there is not a full agreement concerning the origin of the piezoelectric losses. Most authors consider that features in piezoceramic materials that are responsive to both the elastic and electric fields (such as certain point defects in crystals, non-180° ferroelectric-ferroelastic domains, second phase in the piezoelectric polymer materials, *etc.*) are a source of piezoelectric relaxation through the coupling of the elastic and dielectric losses.

Here, to study losses in piezoelectric materials we will take a harmonic stimulus and response. The use of a single harmonic stimulus allows us to use complex numbers for the physical constants. In this case, we separate the in-phase response as the real part of the constant and the in-quadrature response as the imaginary part.

Along this work, we will use the notation recommended in the IEEE Standard on Piezoelectricity [16].

### 1.2. Imaginary Part versus Phase Angle

**Figure 1:**
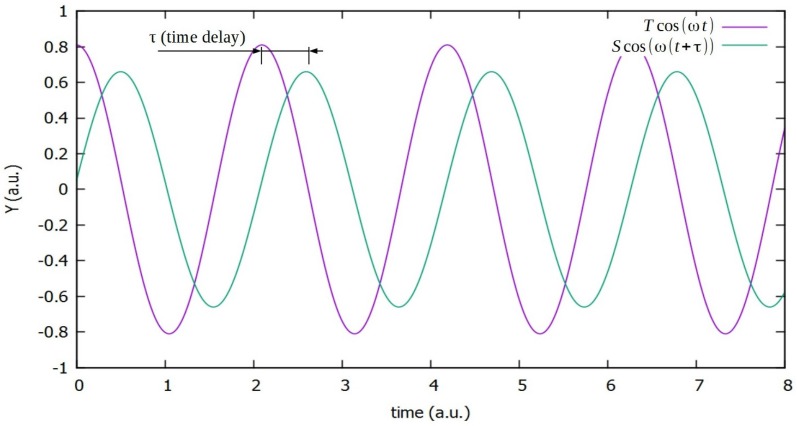
Single harmonic signal and delayed or lossy response.

We will take Hooke’s law for elastic materials under a single harmonic stimulus as an example. In this case, *S* will be the unitary deformation, *T* the strength applied over the specimen, *s* the proportionality factor among them, the compliance of the material and *θ* the phase angle, which will allow us to characterize the time delay between the stimulus and the response (Figure 1). Then, for a material with mechanical losses, we obtain Equation (1):

(1) Scos(ωt+θ)= s T cos(ωt)

where we can expand the left part of the equation as Scosθcosωt+Ssinθsinωt. In order to solve this equation, we will use complex numbers to represent magnitudes and proportionality factors, then $Se^{i\left(\omega t+\theta \right)}=se^{i\theta }Te^{i\omega t}$, where we can write every quantity in the form (*real* + *i imaginary*):

(2) $$\begin{array}{c} Se^{i\left(\omega t+\theta \right)}=S\cos\left(\omega t+\theta \right)+i\;S\sin\left(\omega t+\theta \right)\\ se^{i\theta }=s\cos\theta +i\;s\sin\theta \\ Te^{i\omega t}=T\cos\omega t+i\;T\sin\omega t \end{array}$$

Then, taking the real parts, we found:

(3) Scosθcosωt−Ssinθsinωt=sTcosθcosωt−sTsinθsinωt 

This can be expressed alternatively as:

(4) $$s\;=\left|s\right|e^{i\theta }=s\cos\theta +\;i\;s\sin\theta =s^{\prime }-i\;s\prime \prime$$

As can be seen, we write an explicit minus sign between the real and imaginary parts. This notation allow us to define *s*″ as positive. This convention is the same used for dielectric losses.

To understand this convention, we must rewrite the unitary deformation as:

(5) Scos(ωt+θ)=Scosω(t−τ)

In this way, we use the new variable, *τ*, as the time delay. This time delay must be positive, due to the causality principle, and then, the phase delay becomes θ= −ωτ≤0. Moreover, $\theta \geq \;-\frac{\pi }{2}$, because for lower values than this, the term we find scosθ<0, and the response should be opposite the stimulus. All of this implies:

(6) $$-\frac{\pi }{2}\leq \theta \leq 0,\;and\;s\prime \prime =s\sin\theta \leq 0$$

### 1.3. Dielectric and Mechanical Losses

For a wide range of piezoelectric applications, under adiabatic and linear response conditions, we can use the well-known linear equations of the piezoelectricity:

(7) $$\begin{array}{c} S=s\;T+d\;E\\ D=\epsilon E+dT \end{array}$$

where *S*, *T* and *s* were explained above, *E* and *D* are the electric field and electric displacement, respectively, ϵ is the dielectric permittivity and *d* is the charge piezoelectric constant. All of these parameters must be described by complex numbers, *z*, and hence, we can define their corresponding loss tangent as:

(8) $$\tan\delta_{z}=\;\frac{Im\left(z\right)}{Re\left(z\right)}$$

According to Holland [5], the imaginary parts of the elastic compliance and dielectric permittivity represent the mechanical and electrical losses. Both of them must be explained from the point of view of a delay in the response and may be used to calculate the energy lost as friction in the mechanical equation or as the Joule effect in the electrical one. Then, the three characteristic constants must be written as:

(9) $$\begin{array}{c} \hat{s}=s^{\prime }-\;i\;s\prime \prime \\ \hat{\epsilon }=\;\epsilon^{\prime }-\;i\;\epsilon \prime \prime \\ \hat{d}=s^{\prime }-\;i\;d\prime \prime \end{array}$$

or, as an alternative:

(10) $$\begin{array}{c} \hat{s}=s^{\prime }(1-i\;\tan\delta_{s})\\ \hat{\epsilon }=\epsilon^{\prime }(1-i\;\tan\delta_{\epsilon })\\ \hat{d}=d^{\prime }(1-i\;\tan\delta_{d}) \end{array}$$

where the three imaginary parts, as well as the losses’ tangent are positive numbers.

### 1.4. The Matrix of Coefficients

All of these quantities are tensors (second order for the elastic magnitudes, *S_ij_* and *T_ij_*, and the dielectric permittivity, $\epsilon_{ik}^{T}$, third order for the piezoelectric constant, *d_ijk_*, and forth order for the elastic compliance, $s_{ijkl}^{E}$), and we must rewrite the linear equations as:

(11) $$\begin{array}{c} S_{ij}=s_{ijkl}^{E}T_{kl}+d_{kij}E_{k}\\ D_{i}=d_{ikl}T_{kl}+\epsilon_{ik}^{T}E_{k} \end{array}$$

In order to get the simplest representation of the mechanical constants and magnitudes, we will make the usual index contraction (*i,j* = 1,2,3; *p,q* = 1,…6); thus, $s_{ijkl}^{E}=s_{pq}^{E},\;d_{ijk}=d_{pk}$, and then, the final form for the piezoelectric equations will be:

(12) $$\begin{array}{c} S_{p}=s_{pq}^{E}T_{q}+d_{pk}E_{k}\\ D_{i}=d_{iq}T_{q}+\epsilon_{ik}^{T}E_{k} \end{array}$$

where $S_{ij}=S_{p},\;\left(i=j\to p=1,2,3\right)$ corresponds to extensional stresses along the primary axes and $2S_{ij}=S_{p}\left(i\neq j\to p=4,5,6\right)$ corresponds to shear stresses.

For every crystalline class, due to crystal symmetries, we have different matrices of coefficients. In this work, we will use only those corresponding to electrically-poled piezoceramics (6-mm group of symmetry) [17], which can be used for other kind of materials (such as composites or ferroelectric polymers).

In this case, Direction 3 is assumed as that corresponding to the polar axis, and Directions 1 and 2 are equivalents. Then, for the non-zero coefficients, we find the following relations: $s_{11}=s_{22}$; $s_{13}=s_{23}=s_{31}=s_{32}$; $s_{44}=s_{55}$; $s_{66}=2\left(s_{11}-s_{12}\right);$ $d_{31}=d_{32}$; $d_{15}=d_{24}$; $\epsilon_{11}=\epsilon_{22}$.

Additionally, the whole matrix has only 10 independent elements (five elastic, three piezoelectric and two dielectric coefficients):

(13) $$\left(\begin{array}{c} S_{1}\\ S_{2}\\ S_{3}\\ S_{4}\\ S_{5}\\ S_{6}\\ D_{1}\\ D_{2}\\ D_{3} \end{array}\right)=\;\left(\begin{array}{c} s_{11}\\ s_{12}\\ s_{13}\\ \cdot \\ \cdot \\ \cdot \\ \cdot \\ \cdot \\ d_{31} \end{array}\begin{array}{c} s_{12}\\ s_{11}\\ s_{13}\\ \cdot \\ \cdot \\ \cdot \\ \cdot \\ \cdot \\ d_{31} \end{array}\begin{array}{c} s_{13}\\ s_{13}\\ s_{33}\\ \cdot \\ \cdot \\ \cdot \\ \cdot \\ \cdot \\ d_{33} \end{array}\begin{array}{c} \cdot \\ \cdot \\ \cdot \\ s_{44}\\ \cdot \\ \cdot \\ \cdot \\ d_{15}\\ \cdot \end{array}\begin{array}{c} \cdot \\ \cdot \\ \cdot \\ \cdot \\ s_{44}\\ \cdot \\ d_{15}\\ \cdot \\ \cdot \end{array}\begin{array}{c} \cdot \\ \cdot \\ \cdot \\ \cdot \\ \cdot \\ s_{66}\\ \cdot \\ \cdot \\ \cdot \end{array}\begin{array}{c} \cdot \\ \cdot \\ \cdot \\ \cdot \\ d_{15}\\ \cdot \\ \epsilon_{11}\\ \cdot \\ \cdot \end{array}\begin{array}{c} \cdot \\ \cdot \\ \cdot \\ d_{15}\\ \cdot \\ \cdot \\ \cdot \\ \epsilon_{11}\\ \cdot \end{array}\begin{array}{c} d_{31}\\ d_{31}\\ d_{33}\\ \cdot \\ \cdot \\ \cdot \\ \cdot \\ \cdot \\ \epsilon_{33} \end{array}\right)\left(\begin{array}{c} S_{1}\\ S_{2}\\ S_{3}\\ S_{4}\\ S_{5}\\ S_{6}\\ D_{1}\\ D_{2}\\ D_{3} \end{array}\right)$$

### 1.5. Piezoelectric Losses

We have no reason to assume that the piezoelectric coefficients in the matrix are not complex quantities if both elastic and dielectric coefficients are complex. In fact, Holland [5] probes the necessity of using an imaginary part for the piezoelectric constants and found the limits imposed by thermodynamical considerations.

The simplest explanation is that the imaginary part corresponds to losses during the energy conversion. From the point of view of the piezoelectric coefficient *d*:

(14) $$d=\frac{\partial S}{\partial E}=\frac{\partial D}{\partial T}$$

We can read these losses as a delay between stimulus and response (as we did above) or as a result of crossed interactions between the two forms of energy (frictional losses under electric fields and the increment of the electric resistance under mechanical stresses).

The ratio between energies gets us the efficiency of the mechanical conversion and is called the electromechanical coupling factor, defined as:

(15) $$k^{2}=\frac{W_{piezo}^{2}}{W_{elastic}W_{electric}}$$

where $W_{piezo},\;W_{elastic}\;\text{and}\;W_{electric}$ are their corresponding energies. This factor is strongly dependent on the conditions of the transduction (quasistatic, dynamic) and, of course, the coefficients involved. For dynamical conversion (as those due to a harmonic stimulus), the coupling factor can be found by means of the relation:

(16) $$k_{ip}^{2}=\frac{d_{ip}^{2}}{s_{pq}^{E}\epsilon_{ij}^{T}}$$

We make use of complex quantities to include information about losses. It must be remarked that this works correctly only for single harmonic stimulus and response, as used with resonant methods of characterization, and only for the linear response range. In the rest of the cases, the representation of losses is more difficult, such that we must take into account non-linear effects, intermodulation, coupling between modes, *etc.*, but the fundamental physics of the material losses can be studied under the simplest conditions.

Although the three characteristic constants of the material ($d_{ip},\;s_{pq}^{E}\;\text{and}\;\epsilon_{ij}^{T}$) are independent, there are limits, described by Holland [5], for the values of their imaginary parts. As an example, for the length vibration of a bar poled in this direction, the corresponding constants will be $d_{33},s_{33}^{E},\epsilon_{33}^{T}$, and the condition:

(17) $${\left(d_{33}^{\prime \prime }\right)}^{2}\leq s_{11}^{E}\prime \prime \cdot \epsilon_{33}^{T}\prime \prime$$

Even more, the constants $s_{33}$ and $\epsilon_{33}$ are different before and after the resonance and are related by the equations:

(18) $$\begin{array}{c} s_{33}^{D}=s_{33}^{E}\left(1-k_{33}^{2}\right)\\ \epsilon_{ij}^{S}=\epsilon_{ij}^{T}\left(1-k_{33}^{2}\right) \end{array}$$

where superscripts *E* and *T* correspond to “free” conditions (frequencies below that the resonance) and *D* and *T* correspond to “clamped” conditions (above the resonance). That means that for the relation between the three constants, in this case, the clamped compliance includes not only the mechanical response, but the interaction with the dielectric and piezoelectric one. We find the same behavior for the permittivity that includes the mechanical and piezoelectric responses. Their respective losses will be, of course, related.

Noticeably, the fact that piezoelectric coefficients of piezo materials have a complex form indicates the presence of extrinsic contributions to the piezoelectricity in these materials, regardless of the actual mechanisms involved [18].

Piezoelectric losses are the most controversial [13,19] and difficult to measure independently, because when the material deforms under the action of the piezoelectric effect, it also must endure mechanical loss. The hysteresis is also indicative of the loss. Alternatively to the resonance method, a number of methods were proposed for the measurement of the phase angle between strain and field [14,18]. These alternative measurements [14] yield piezoelectric loss values in the linear range that are in good agreement with iterative methods at the resonance [20,21].

## 2. Material Characterization from Electromechanical Resonances

One of the most common ways to obtain an easy and accurate characterization is the resonant method. It consists of getting the complex impedance spectrum of a resonator, including at least one electromechanical resonance. We consider the material sample as a resonant cavity (a propagation media and its boundaries). Knowing the geometrical factors of the resonant cavity and a mathematical model for the impedance function that takes into account this resonance, we can obtain the elastic, piezoelectric and dielectric coefficients involved in this particular resonance. For reasons explained later, in some cases, the description in terms of admittance is easier than the impedance representation. We must, of course, use the complex form for both, that is: Z=R+i X and Y=G+i B.

The most general function describing the impedance of a sample shape as a thin plate in the neighborhood of a resonance is:

(19) Z=1iωϵSℓ(1−k2F(ω,s)) 

where *S* is the area of the electrodes, ℓ the distance between them, ω the angular frequency and ϵ the dielectric permittivity. The dielectric capacitance of the sample will be C=ϵ S/ℓ, then we can observe that this relation corresponds to the characteristic impedance of a capacitor, $Z={\left(i\omega C\right)}^{-1}$, modified by the resonant term, $k^{2}F\left(\omega ,s\right)$. The electromechanical coupling factor, $k^{2}$, was previously defined, and the function F(ω,s) depends on the shape of the sample and the speed of the sound across the material (that implies a dependence on the elastic coefficient) and is responsible for the resonance.

### 2.1. Shapes and Modes

As we treat the sample as a resonant cavity, shape and dimension are the most important parameters to take into account for the study of the standing waves inside and getting information from them of the material.

The set of linear equations of the piezoelectricity is written under the assumption of the existence of an external force and an electric field (by means of a voltage in electrodes). Nevertheless, depending on the contour conditions, we can rewrite such linear equations as:

(20) $$\begin{array}{c} \left(\begin{array}{c} T\\ D \end{array}\right)=\left(\begin{array}{cc} c^{E} & e\\ e & \epsilon^{S} \end{array}\right)\;\left(\begin{array}{c} S\\ E \end{array}\right),\;\left(\begin{array}{c} S\\ D \end{array}\right)=\left(\begin{array}{cc} s^{E} & d\\ d & \epsilon^{T} \end{array}\right)\;\left(\begin{array}{c} T\\ E \end{array}\right)\;\\ \left(\begin{array}{c} S\\ E \end{array}\right)=\left(\begin{array}{cc} s^{D} & g\\ -g & {\left(\epsilon^{T}\right)}^{-1} \end{array}\right)\;\left(\begin{array}{c} T\\ D \end{array}\right),\;\left(\begin{array}{c} T\\ E \end{array}\right)=\left(\begin{array}{cc} c^{D} & h\\ -h & {\left(\epsilon^{S}\right)}^{-1} \end{array}\right)\;\left(\begin{array}{c} S\\ D \end{array}\right) \end{array}$$

Then, we find five new constants: one mechanical, *c*, the stiffness, one dielectric, β, that we prefer to write as $\epsilon^{-1}$, and three piezoelectric constants, *g* (called voltage piezoelectric constant), *h* and *e*.

### 2.2. The Iterative Method

To get a complete characterization of a piezoceramic, we must get a set of 10 different parameters, the independent elements of the characteristic matrix. Since these materials exhibit non-negligible losses, it is needed to use alternative characterization methods to that proposed by the current standards [16], which do not account for all losses. Numerous authors have developed such alternative modes, and the interested reader can find reference to them in [22]. New methods keep on being published on this topic; among others, those based on the iterative modification of finite elements simulations to match measurements in the best possible way are noticeable [23,24].

For determining these elements in a given resonant mode, we propose here Alemany’s method [20,25] and the available free-ware to use it [26]. This fully-automatic iterative method, based on Smits´ one [27], solves numerically the analytical expression for the wave equation of a given mode of resonance to obtain the parameters involved in it. Typically, the directly calculated parameters from a resonance mode are one elastic, one dielectric and one piezoelectric parameter (except for the radial mode of disks, which provides two elastic constants). The selection, based on the standards for measurements [16], of three resonator shapes and four modes of resonance, together with the smart combination of the parameters directly calculated from these analytical expressions with the remaining parameters, allow us to get the whole set of complex material parameters [28]. This method of analysis of the complex impedance curves has been also applied to the determination of the parameters from overtone resonances in the radial [29] and thickness [30] modes of thin disks to account for the frequency dependence of these parameters. Besides, the principles for the application of this method to the determination of the properties of self-standing films were developed and applied to lead zirconate titanate (PZT) cantilevers [31].

**Figure 2:**
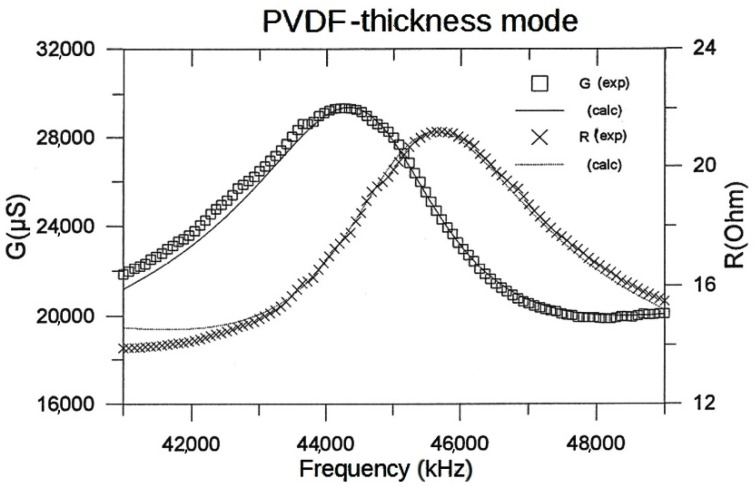
Example of the characterization of a piezoelectric polymer (PVDF: polyvinylidene fluoride).

Using Alemany’s iterative method, we get the complex values of every parameter and, then, information about losses and the relations between them. This procedure is not limited to piezoceramics, and we used it for the characterization of ferroelectric polymers (Figure 2) and composites.

The main limit of the resonance method consists of the coupling between modes. This problem cannot be solved as suggested by the standard IEEE-176 [16], because there is no a simple superposition of modes, but a coupling with energy exchange between them.

The impedance spectra of the shear plates used in standard characterization methods from resonance, in-plane poled and excited in thickness, always show additional peaks, satellite resonances, around that of the main resonance. These correspond to natural modes of vibration of plates, such as contour modes, and are unavoidable, since they are excited simultaneously to the shear mode. When using an alternative shear geometry [32], thickness poled and in-plane excited, it is possible, by tuning the aspect ratio of the sample (Length and width to thickness = *L*,*w*/*t*), through the reduction of the thickness, to obtain uncoupled shear modes in a periodical way, even below the standard ratio of *L*,*w*/*t* = 10. Accurate values of impedance around resonance and anti-resonance frequencies are required to determine accurately the material parameters, including losses, which can only be obtained from uncoupled modes. For the optimum sample aspect ratios obtained in the first four periods, the dispersion in the so-measured parameters are 0.4% for the k_15_ coupling factor and for the directly-calculated parameters, $s_{55}^{E}$, $e_{15}$ and $\epsilon_{11}^{S}$, less than 2%, in their real part, and 10%, 12% and 13% in their imaginary part, respectively. This is a unique result to the authors’ knowledge, given the difficulties encountered to measure even the real part of this coefficient by other methods [33]. An additional advantage of the use of the thickness poled shear plate for the full characterization of the piezoceramics is that, since it can be obtained from the disk after measuring it, in principle, this will allow the characterization from two resonators, a thin disk, thickness poled and a long rod or bar, length poled [34]. Finally, the higher consistency with the parameters is obtained from the thickness poled disk, since for both resonators, the spatial distribution of the polarization is identical. Due to this enhanced consistency, a finite element modelling, based on the full matrix characterization using thickness poled shear plates, was successfully tested for both the impedance spectra and displacement patterns. Good agreement with the experimental, electrical and laser interferometry measurements, respectively, was achieved in a range between 325 kHz and 1.2 MHz.

### 2.3. Plotting Data

There are no standards for graphing the piezoelectric characterization in resonant mode. Most of the time, researchers represent as-measured data from the impedance analyzer, *i.e.*, the absolute value, |*Z*|, and the argument, $\theta_{Z}$, of the impedance. Using this plot, we can get a good idea about the electric anti-resonance, which is clearly shown by the maximum electrical impedance.

For some vibrational modes, as thickness extensional modes, where the direction of the electrical excitation and movement are parallel, due to the boundary conditions, the mechanical resonance corresponds to the maximum of the impedance, *i.e.*, the electric anti-resonance. However, for modes where the mechanical movement is perpendicular to the direction of the electrical excitation, e.g., length expansion of thin bars thickness poled or radial modes of thickness poled thin discs, the mechanical resonance corresponds to the minimum of the electrical impedance, *i.e.*, the electrical resonance. This is due to the fact that the electric open/short circuit conditions are interchanged with the mechanical ones.

We use the frequency of the mechanical resonance to determine the complex mechanical compliance, *s*, or stiffness, *c*:

(21) $$s_{ij}^{E}=\frac{1}{4\rho \ell^{2}f_{s}^{2}}\left(1-i\frac{\Delta f_{s}}{f_{s}}\right),\;or\;c_{ij}=4\rho t^{2}f_{p}^{2}\left(1-i\frac{\Delta f_{p}}{f_{p}}\right)$$

These frequencies do not correspond to the maximum or minimum of the *Z*-plot (usually called: $f_{n},\;f_{m}$), which give us wrong values for this purpose in lossy materials, and we must use instead the maxima of the real parts of admittance and impedance ($f_{s},\;f_{p}$) [16].

**Figure 3:**
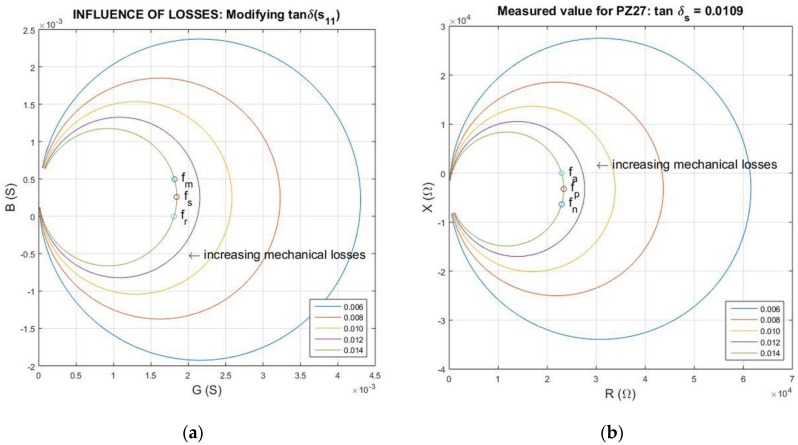
(**a**) *G-B* and (**b**) *R-X* plots showing the “position” of the three possible resonance frequencies (in the *G-B* plot): *f_r_*, zero reactance; *f_s_*, maximum conductance; *f_m_*, maximum absolute admittance; and the three possible anti-resonance frequencies (at *R-X* plot): *f_a_*, zero susceptance; *f_p_*, maximum resistance; *f_n_*, maximum absolute impedance.

There is another pair of resonance-anti-resonance frequencies; those correspond to the zeros for the values of the susceptance or reactance: $f_{r},\;f_{a}$, respectively. In Figure 3, the three possible anti-resonance frequencies are shown in admittance-*GB* and impedance-*RX* plots.

For lossless resonators, these frequencies are close, but the difference is very important for lossy and/or weak resonators.

**Figure 4:**
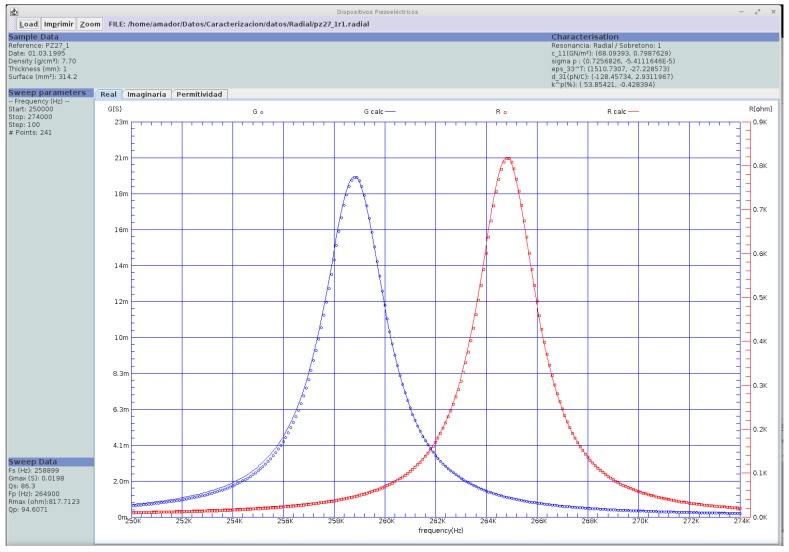
Presentation of the results obtained using Alemany’s method for measurement at the first overtone of the radial resonance mode of a PZ27 (Ferroperm Piezoceramics A/S) thin disc.

The authors currently use a dual plot that includes the real parts of the impedance (conductance, *G*) and the admittance (resistance, *R*) that includes both peaks (Figure 4). Alemany’s [20,25] method uses information from anti-resonance to increase the accuracy in the characterization, including all losses.

The display of the Java version for the Alemany’s method software is shown in Figure 4. The experimental data and the reproduced spectrum for the first overtone of the radial mode of a PZ27 (Ferroperm Piezoceramics A/S) thin disc of *t* = 1 mm and *D* = 20 mm are plotted [25]. The reproduction is carried out by introducing the calculated material parameters back into the analytical expression used for the iterative numerical solution of the impedance measurement:

(22) $$Y=i\omega \frac{S}{t}\epsilon_{33}^{T}\left(1-\frac{k_{p}^{2}}{\frac{1}{2-J\left(\xi \right)}-\;\frac{1}{1+\sigma^{p}}}\right)$$

where ω is the driven frequency, *S* the area of electrodes, *t* the thickness, $\epsilon_{33}^{T}$ the dielectric permittivity at constant stress, *k_p_* the planar coupling factor, $\sigma =-s_{12}^{E}/s_{11}^{E}$ Poisson’s planar ratio and J(ξ) the so-called Onoe’ s function, defined by:

(23) $$J\left(\xi \right)=\;\frac{\xi \;J_{0}\left(\xi \right)}{J_{1}\left(\xi \right)},\;\text{with }\xi =\omega a\sqrt{\frac{c_{11}^{p}}{\rho }}\;$$

where *J*_0_ and *J*_1_ are Bessel functions of the first kind, and of zeroth and first order, respectively, *a* is the radius of the disc, ρ the density and $c_{11}^{p}=s_{11}^{E}/\left[{\left(s_{11}^{E}\right)}^{2}-{\left(s_{12}^{E}\right)}^{2}\right]$ the stiffness of the planar mode.

As that is a monomodal resonance, there is a perfect agreement between the measured and the reproduced spectra.

## 3. Studying Losses

### 3.1. What Losses Look Like?

For a piezoelectric resonator, as we mentioned above, it can be expressed as the loss tangent: tanδ , but also using its inverse, the *Q*-factor, defined as:

(24) $$\tan\delta =\frac{1}{Q}=\;\frac{\Delta f}{f_{0}}$$

where *f_0_* is the resonant frequency and Δ*f* is the FWHM (full with at half maximum). For a pure mechanical resonator, the loss tangent of the resonator corresponds to those of the elastic constant, and then, this is equivalent to:

(25) $$\tan\delta =\;\frac{s\prime \prime }{s\prime }=-\frac{c\prime \prime }{c\prime }$$

with *s*’, *s*” as the real and imaginary parts of the compliance and *c*’, *c*” the real and imaginary parts of the stiffness.

This loss tangent can be assigned to both resonance and anti-resonance peaks. As mentioned above, the lowest frequency, the so-called “free” elastic stiffness, $c_{pq}^{E}$, is that corresponding to constant *E* conditions, and the higher frequency, the “clamped” stiffness, $c_{pq}^{D}$, corresponds to constant *D*.

**Figure 5:**
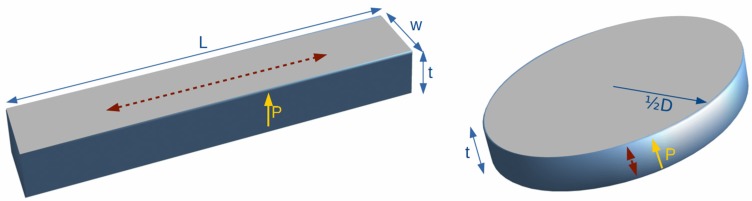
The two resonator geometries used in the simulation of the effect of the losses on the resonance.

In the following, we will study the influence of the different loss factors on the behavior of the sample at the electromechanical resonance, *i.e.*, in the frequency range between the resonance and the anti-resonance frequencies, of two piezoceramic resonator geometries of the same material, Ferroperm A/S PZ27, a Navy type II piezoceramic (Figure 5). One geometry is a thickness poled rectangular bar (*L* = 31.3 mm, *w* = 1.46 mm and *t* = 0.65 mm) at the length extensional resonance mode; the other one is a thickness poled thin disc (*t* = 1 mm and *D* = 20 mm) at the thickness mode. Both of them are electrically excited in thickness. Experimental measurements on these samples and the relevant complex material parameters (Table 1) calculated from them using Alemany’s [20,29] method were used as a starting point of the simulations.

**Table 1:** Experimental characterization parameters obtained by Alemany’s method for two samples of PZ27 piezoceramic.

Shape	*Rod*
mode	*length extensional*
Thickness (m)	0.65 × 10^–3^
length (m)	31.3 × 10^–3^
width (m)	1.46 × 10^–3^
density (${\text{kg m}}^{-3}$)	7700
$\epsilon_{33}^{T}$	$\left(1682-i\;25\right)\epsilon_{0}$
$s_{11}^{E}\;(m^{2}{\text{ N}}^{-1})$	$\left(16.011-i\;0.175\right)10^{-12}$
$d_{31}\left({\text{C N}}^{-1}\right)$	$\left(-159.22+i\;3.0\right)10^{-12}$
$k_{31}$	0.3259 + i 0.0103
**Shape**	***Disc***
mode	*thickness extensional*
Thickness (m)	1.00 × 10^–3^
radius (m)	20.0 × 10^–3^
density $\left({\text{kg m}}^{-3}\right)$	7700
$\epsilon_{33}^{S}$	$\left(818.6-i\;23.9\right)\epsilon_{0}$
$c_{33}^{D}\;\left({\text{N m}}^{-2}\right)$	$\left(136.9\;+\;i\;0.i\right)10^{9}$
$h_{33}\;\left(V\;m^{-1}\right)$	$\left(1.995\;+i\;0.036\right)10^{9}$
$k_{t}$	0.4592+ i 0.0004

The spectra were reproduced from these material parameters using MATLAB^®^ R2015a software and the corresponding analytical solution of the wave equation for each considered resonance mode [20,29]. Then, the losses were both increased and decreased from that experimental reference, modifying the imaginary part of one specific constant each time (elastic, dielectric and piezoelectric). The following Figures show the so-obtained parameterized spectra.

### 3.2. Influence of the Mechanical Losses

**Figure 6:**
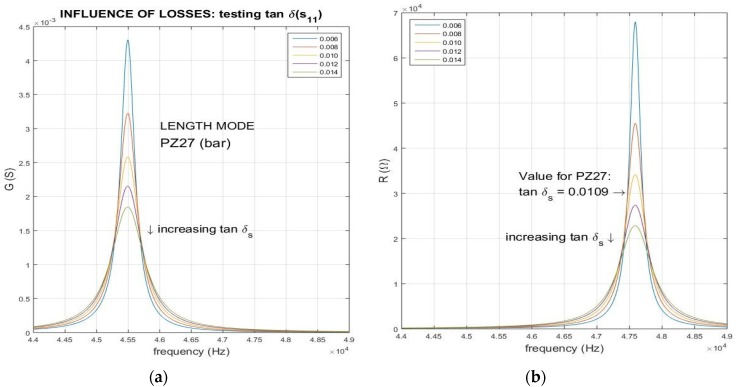

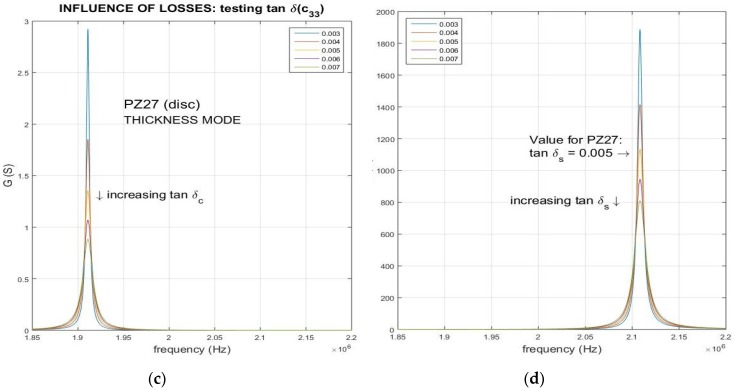
Influence of mechanical losses on the (**a**) and (**c**) resonance G-peaks; and (**b**) and (**d**) anti-resonance R-peaks. The increment of the losses of the elastic parameter (compliance) affects both peaks in the same way.

The effect produced by the change of the elastic constant loss tangent is shown in Figure 6. In this case, we show the resonance-anti-resonance peaks of the length extensional mode of a bar, and then, we use the elastic compliance $s_{11}^{E}$ and simulate the effect of varying losses around the measured one ($\tan\delta \left(s_{11}^{E}\right)=0.0109$). Both peaks change simultaneously and get wider with the increase of the losses, as expected. This is the classical result: low-Q (high mechanical loss) resonators presents wide peaks.

As we mentioned above, for the radial mode of resonance of thin disks, it is necessary take into account two mechanical constants, $c_{11}^{P}$ and $\sigma^{P}$, which represent the contour stiffness constant and the planar Poisson modulus, and those are related to the most common compliances $s_{11}^{E},\;s_{12}^{E}$ by means of the relations:

(26) $$\begin{array}{c} c_{11}^{P}=\frac{s_{11}^{E}}{{\left(s_{11}^{E}\right)}^{2}-{\left(s_{12}^{E}\right)}^{2}}\\ \sigma^{P}=-s_{12}^{E}/s_{11\;}^{E} \end{array}$$

In this case, the imaginary part of $\sigma^{P}$ seems to be null within the experimental errors in all materials that we studied, thus meaning that $\tan\;\delta \left(s_{11}^{E}\right)=\tan\delta \left(s_{12}^{E}\right)$, and then:

(27) $$\tan\delta =-\frac{s_{11}^{E}\prime \prime }{s_{11}^{E}\prime }=\frac{\left(c_{11}^{P}\right)\prime \prime }{\left(c_{11}^{P}\right)\prime }$$

### 3.3. Influence of the Dielectric Losses

**Figure 7:**
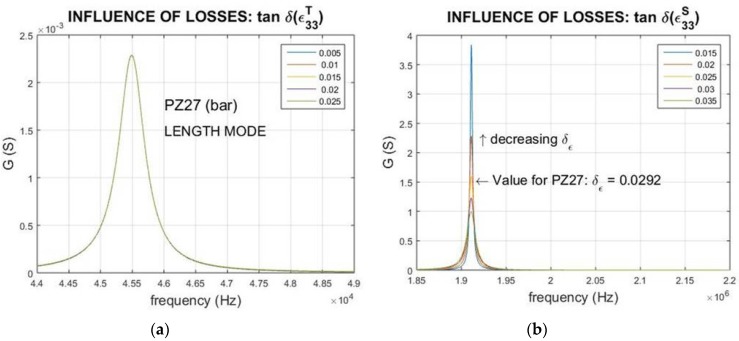

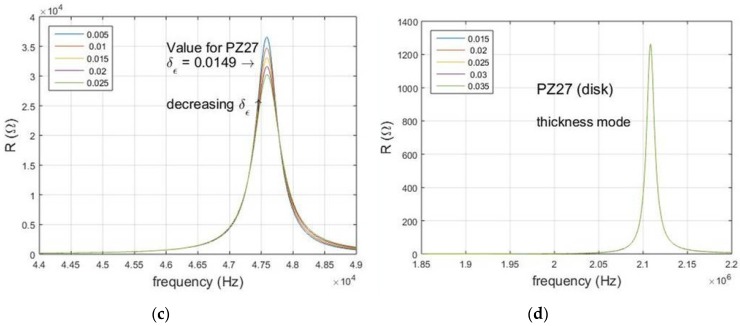
Influence of the dielectric losses on the (**a**) resonance and (**c**) anti-resonance peaks for length extensional mode of a thickness poled bar; dielectric losses modify only (see the *R*-peak) the elastic anti-resonance peak (also electric anti-resonance). Dielectric losses influence on the (**b**) resonance and (**d**) anti-resonance for thickness extensional mode of a thin disc, thickness poled; dielectric losses modify the elastic anti-resonance, which in this case corresponds to the *G*-peak (electric resonance).

The influence of modifying the loss factor of the dielectric permittivity is shown in Figure 7. Note the increasing of the width in the peak corresponding to the mechanical anti-resonance with the increase of the dielectric losses, while the mechanical resonance peak remains unchanged. For the thickness poled bar vibrating along its longest dimension, the mechanical anti-resonance corresponds to the electric one; thus, the *R*-peak changes.

For the thin disc, thickness poled, in the thickness resonance, the mechanical anti-resonance corresponds to the electric resonance, in this case the *G*-peak exhibits variation in its width.

Then, the effect of the dielectric losses is observed only in the mechanical anti-resonance getting a lower value of the *Q*-factor with the increase of the dielectric losses.

### 3.4. Influence of the Piezoelectric Losses (Piezoelectric Modulus)

**Figure 8:**
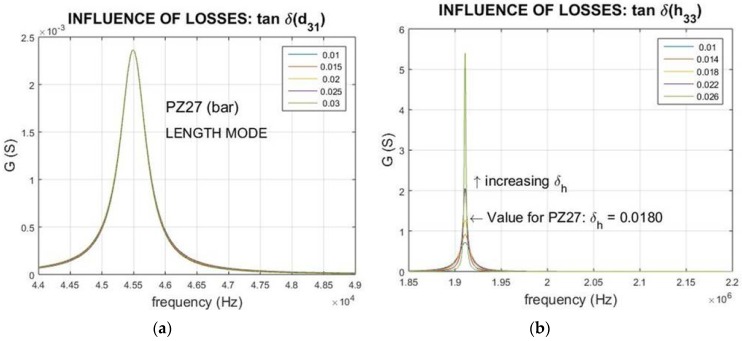

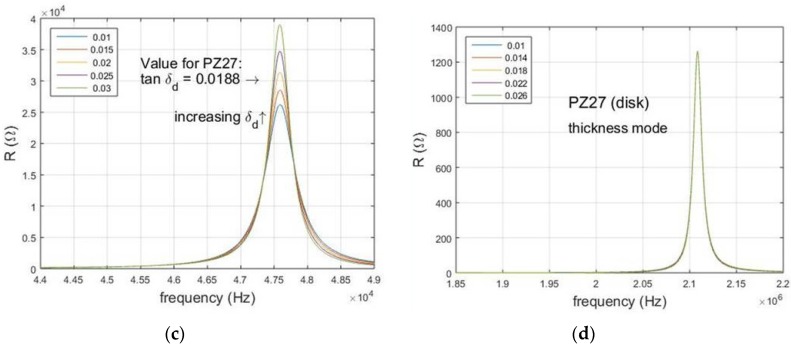
Influence of piezoelectric losses on the (**a**) resonance and (**c**) anti-resonance peaks for length extensional mode of a thickness poled bar; loss of *d*_31_ modifies the mechanical anti-resonance peak (*R*-peak). Influence of piezoelectric losses on the (**b**) resonance and (**d**) anti-resonance peaks for thickness mode of a thin disc, the losses of the *h*_33_ factor modify the mechanical anti-resonance peak (*G*-plot).

The influence of the piezoelectric losses, while keeping both mechanical and dielectric losses constant, is shown in Figure 8.

Here, the simulation of small variations around the measured value for the imaginary part of the piezoelectric constant involved in every resonant mode ($d_{31}$ for the length extensional mode of the bar and $h_{33}$ for the thickness mode of the thin disk) is carried out. It is noticeable that their influence is different from that observed in the two previous simulations. As for the dielectric losses, for both studied resonances, the change of piezoelectric losses only affects the mechanical anti-resonance. However, increasing losses in this case produce narrower peaks (getting a higher *Q*-factor that corresponding to the resonance).

This anisotropy of loss factors (resonance and anti-resonance) is observed in materials with high piezoelectric losses (also reported by Uchino [10]), but could be masked by the opposite effect of the dielectric losses; thus, an accurate method is necessary to get the correct characterization.

## 4. Losses in Devices

Piezoelectric materials are developed to be integrated in a device or system. It is possible to characterize devices instead of materials [35], but it is not so easy to use this information to design a device.

On the one hand, electrical engineers developed toolboxes (such as SPICE: Simulation Program with Integrated Circuit Emphasis) to make the design and implementation of a system easier by means of equivalent circuits, but if we want to include a piezoelectric resonator in their databanks we need to speak a common language, *i.e.*, we must give the equivalent circuit of our element.

On the other hand, from early times of the study of piezoelectric materials, the strategies to determine the electric components of the equivalent circuit of a piezoelectric resonator from electrical measurements around an electromechanical resonance were a matter of concern [36].

**Figure 9:**
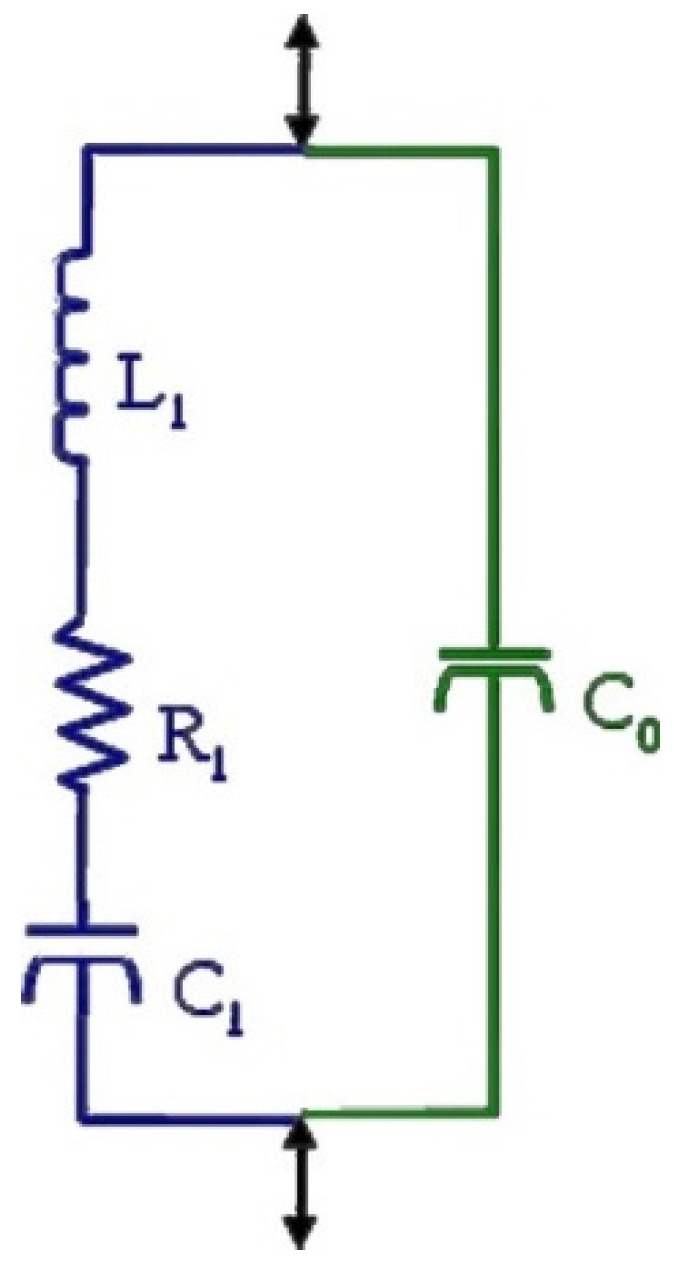
Elementary model of a piezoelectric resonator based on passive elements.

In the beginning, standards proposed the use of the simplest resonant circuit (Figure 9), but do not provide a proper resonance peak shape and describe only the fundamental resonance or one of its overtones. Besides, the mechanical losses are represented by the resistance in the “motional” LCR branch; the dielectric losses must be accounted for by additional series and parallel resistances, but piezoelectric losses require complex components.

More accurate models have been developed and tested for electromechanical transducers [37,38,39], but typically, if one can get from them a higher accuracy, this is at the expense of the increasing complexity of the model and the use of non-linear or more sophisticated elements as transformers.

As a general consideration, a model should not be more complex than the object that it represents.

**Figure 10:**
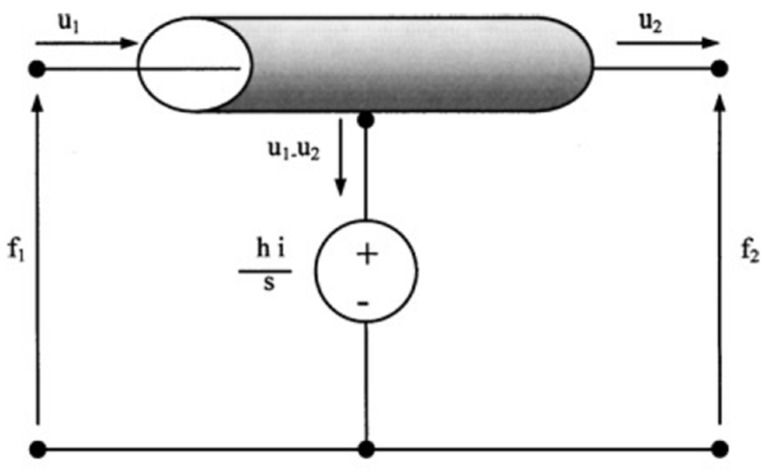
Analogous circuit based on a transmission line for the thickness-mode transducer.

In this work, we introduce a simplified alternative equivalent circuit model [40], given by the use of a transmission line as one of its components (Figure 10) due to the analogies between the distributed elements of the transmission line and the media through which the acoustic waves propagate [41].

**Figure 11:**
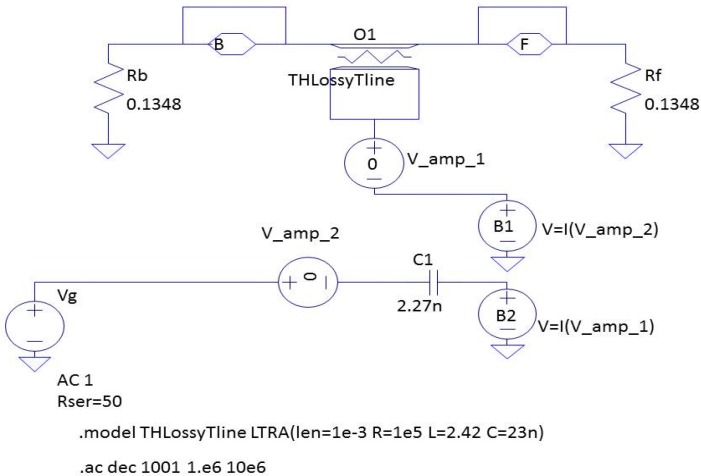
PSPICE schematic, which describes the equivalent circuit of a given device (under simulation) using a single piezoelectric element, represented by the lossy transmission line.

**Figure 12:**
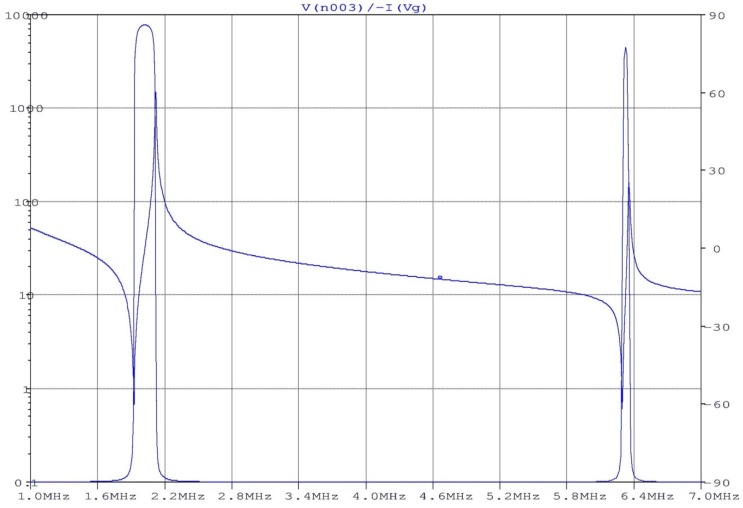
Plot of the modelled impedance of a device using a single piezoelectric element.

Using a model like Püttmer’s [42], mechanical and electrical losses can be easily introduced to model a low-*Q* piezoelectric resonator. Figure 11 shows the description of this model using ORCAD/PSPICE. The figure depicts the different circuit elements modeling the electrical behavior of the piezoelectric material once integrated into the full device [43]. This example corresponds to a simple device, an ultrasonic emitter and receiver, using a single piezoelectric element. Figure 12 draws the impedance (modulus and phase) as a function of the frequency resulting from the device simulation. The use of the lossy transmission line is appropriate for simulating both the multiple resonances and losses. It is worth noting that the results obtained by using the proposed simulation model reproduce the overtones of the resonances. Dependent generators in the model allow simulating mode coupling. This simple model can be extended to account separately for the piezoelectric losses from an accurate material characterization. Work is in progress to this aim.

## 5. Conclusions

Fundamentals of the losses in piezoelectric materials were reviewed. The analysis of the influence of each type of loss (dielectric, elastic and piezoelectric) on the resonance and anti-resonance peaks for resonators with the acoustic wave parallel and perpendicular to the electrical excitation was carried on. Analysis reveals that each kind of loss has a characteristic effect. To connect the field of material researchers with electrical and electronic engineers we proposed, on the one hand, the use of accurate methods of characterization from impedance measurements at resonance and, on the other hand, simplified equivalent circuit models using transmission lines.
